# Crosstalk between the aging intestinal microflora and the brain in ischemic stroke

**DOI:** 10.3389/fnagi.2022.998049

**Published:** 2022-10-06

**Authors:** Ryszard Pluta, Mirosław Jabłoński, Sławomir Januszewski, Stanisław J. Czuczwar

**Affiliations:** ^1^Laboratory of Ischemic and Neurodegenerative Brain Research, Mossakowski Medical Research Institute, Polish Academy of Sciences, Warsaw, Poland; ^2^Department of Rehabilitation and Orthopedics, Medical University of Lublin, Lublin, Poland; ^3^Department of Pathophysiology, Medical University of Lublin, Lublin, Poland

**Keywords:** aging, gut-brain axis, stroke, brain ischemia, amyloid, tau protein, microglia, astrocytes

## Abstract

Aging is an inevitable phenomenon experienced by animals and humans, and its intensity varies from one individual to another. Aging has been identified as a risk factor for neurodegenerative disorders by influencing the composition of the gut microbiota, microglia activity and cognitive performance. The microbiota-gut-brain axis is a two-way communication path between the gut microbes and the host brain. The aging intestinal microbiota communicates with the brain through secreted metabolites (neurotransmitters), and this phenomenon leads to the destruction of neuronal cells. Numerous external factors, such as living conditions and internal factors related to the age of the host, affect the condition of the intestinal microflora in the form of dysbiosis. Dysbiosis is defined as changes in the composition and function of the gut microflora that affect the pathogenesis, progress, and response to treatment of a disease entity. Dysbiosis occurs when changes in the composition and function of the microbiota exceed the ability of the microflora and its host to restore equilibrium. Dysbiosis leading to dysfunction of the microbiota-gut-brain axis regulates the development and functioning of the host’s nervous, immune, and metabolic systems. Dysbiosis, which causes disturbances in the microbiota-gut-brain axis, is seen with age and with the onset of stroke, and is closely related to the development of risk factors for stroke. The review presents and summarizes the basic elements of the microbiota-gut-brain axis to better understand age-related changes in signaling along the microbiota-gut-brain axis and its dysfunction after stroke. We focused on the relationship between the microbiota-gut-brain axis and aging, emphasizing that all elements of the microbiota-gut-brain axis are subject to age-related changes. We also discuss the interaction between microbiota, microglia and neurons in the aged individuals in the brain after ischemic stroke. Finally, we presented preclinical and clinical studies on the role of the aged microbiota-gut-brain axis in the development of risk factors for stroke and changes in the post-stroke microflora.

## Introduction

Aging is defined as the time-related diminution and disappearance of the physical activities that all animals and humans on Earth pass through. With the development of modern society, people in both Eastern and Western communities are contemplating finding a way to live longer. In recent decades, due to the improvement of living conditions and the increase in living standards in developed and developing countries, the life span of people has significantly increased. As a consequence, the number of people aging is much greater than before, and the percentage of people over 60 will rise to 22% by the end of 2050 (Zhou et al., 2022). Life expectancy is increasing worldwide and is expected to further improve in most countries beyond 80 by 2040 (Foreman et al., 2018; Bleve et al., 2022). This means a potential increase in the morbidity and prevalence of aging-related diseases, including cardiovascular, musculoskeletal, oncological, and neurological diseases, such as ischemic stroke (Foreman et al., 2018; Bleve et al., 2022). Due to the progressive limitation of physical activity during aging, the human body undergoes a number of pathological changes, such as genomic and proteomic instability, mitochondrial dysfunction, or inflammatory processes (Green et al., 2011; Moskalev et al., 2013; Zhou et al., 2022). Moreover, in today’s society with an inactive lifestyle and an unhealthy diet, the number of diabetics among aging people has increased dramatically with obesity, which has a significant impact on the incidence of strokes (Popa-Wagner et al., 2020). Lifestyle, including addiction to tobacco and alcohol, high-sugar, and fatty diets, as well as some of the internal factors presented above, negatively affect the occurrence, progression, severity, and duration of neurodegenerative changes after stroke (Popa-Wagner et al., 2020).

Metabolic, molecular and structural changes also occur in the brain with age (Fjell and Walhovd, 2010). The hallmarks of an aging brain: are deregulated metabolic state, impaired activity of the neural network, dysregulated activity of neuroglial cells, depletion of stem cells, loss of neurons and cognitive impairment (Mattson and Arumugam, 2018; Baldwin and Greenwood, 2020; Honarpisheh et al., 2022; Zhou et al., 2022). These accelerated phenomena have cognitive consequences in the elderly, which can lead to neurodegenerative diseases such as Alzheimer’s disease and post-stroke brain injury (Fjell and Walhovd, 2010; Baldwin and Greenwood, 2020; Honarpisheh et al., 2022; Zhou et al., 2022). Age-related cognitive decline is a risk factor for developing dementia, depriving the elderly of wellbeing and shortening their lives (Baldwin and Greenwood, 2020).

Recent investigations have shown that changes in the gut microflora in the elderly are associated with neurodegenerative disorders (Honarpisheh et al., 2022; Zhou et al., 2022). The gut-brain axis shows tight connections between the gut and the brain, which is important for the maturation and function of the microglia (Erny et al., 2015; Honarpisheh et al., 2022). Microglia is a group of neuroglial cells that make about 15% of all brain cells (Zhou et al., 2022). As resident macrophage cells, microglia functions as the primary immune defense in the brain. In order to maintain brain homeostasis, the microglia continuously oversees the brain’s microenvironment through its connections with neighboring cells and many factors (Li et al., 2018). During aging, microglia changes from a resting state to an active state and, together with astrocytes, contributes to the development of neurodegenerative diseases (Johnson et al., 2020). Activated microglial cells act in amoeboid stage, produce pro-inflammatory cytokines, blocking amyloid clearance, and are involved in regulating blood-brain barrier integrity and synaptic plasticity in the older brain (Daria et al., 2017; Yousef et al., 2019; Nguyen et al., 2020). These findings point to a new way to slow and even reverse cognitive aging by influencing the microbiota-microglia cooperation. In this review, we will look at the changes in the gut microflora in the elderly, show the age-related microglia changes, and discuss role of neuroinflammation in an aging brain. Next, we will summarize the influence of microflora on microglia behavior in the aging brain and highlight the importance of the microbiota-microglia relationship in neurodegenerative disorders. This knowledge will certainly enrich our understanding of the relationship between age-related cognitive decline and the microbiota-microglia cooperation, pointing to a possible impact on age-related cognitive decline.

## Aging

Due to limited research, little is currently known about the changes in the gut-brain axis with age (Spychala et al., 2018). Aging is associated with loss of nutrient absorption, malnutrition, anorexia, and weakness (Moss et al., 2012). Aging has been shown to be associated with impairment of the intestinal epithelial barrier, loss of intestinal neurons, and altered immune function of the mucosa, resulting in an imbalance in the secretion of pro-inflammatory cytokines (Durgan et al., 2019). Such changes, often referred to as “inflammaging,” can reduce the ability of the elderly to cope with toxic, antigenic, physical and ischemic stress (Durgan et al., 2019). A review of eighteen studies concluded that aging, inflammation, and different microbial compositions may unequivocally contribute to the development of ischemic stroke (Lee et al., 2021). Older mice with a high *Firmicutes*/*Bacteroidetes* ratio were documented to have a higher risk of ischemic stroke and were unable to recover from neurological deficits, contributing to higher mortality (Spychala et al., 2018; Sinagra et al., 2021). Aging increases the risk of neurodegenerative, cerebrovascular and cardiovascular diseases and cancer. Mechanisms underlying biological aging include increased DNA damage, impaired autophagy, telomere shortening, and immunesenescence (Lejault et al., 2020; Conway et al., 2022). More recently, gut microbiota has emerged as a key mediator of aging and age-related pathologies.

## Aging and intestinal homeostasis

The function of the intestinal barrier deteriorates with age, which is associated with an increased incidence of gastrointestinal infections in the advanced years (Qi et al., 2017). Loss of barrier normal function is due to a decrease in tight junction proteins and age-related mucus production which are regulated by the microbiota (Parrish, 2017). An example of this is *Akkermansia muciniphila* which is lower in patients with age-related diseases than in healthy people of the same age (Singh et al., 2019). *Akkermansia muciniphila* supplementation relieves the age-related reduction in the thickness of the colon mucus (van der Lugt et al., 2019). A probiotic cocktail of human origin isolated from healthy infants, administered to older mice with dysbiosis reduced intestinal permeability by increased activity of bile salt hydrolase and by modulation of the microbiota/taurine/tight junction axis (Ahmadi et al., 2020). These observations suggest that age-related intestinal barrier dysfunction can be prevented by using microbiota-based therapies.

Secretion of interleukin-22, which stimulates the regenerative abilities of crypt stem cells, decreases with age (Lindemans et al., 2015; de Haan and Lazare, 2018). Generation of interleukin-22 by innate intestinal lymphoid cells is induced by microbial metabolites such as indole-3-carboxaldehyde and aryl hydrocarbon receptor ligand derived from tryptophan (Zelante et al., 2013; Lindemans et al., 2015). The ability of the microflora to synthesize indoles gradually decreases with age (Ruiz-Ruiz et al., 2020). These observations indicate that the deficiency of indole-based metabolites noted with age reduces the production of interleukin-22 dependent on innate lymphoid cells, which limits the regenerative capacity of crypt stem cells (Honarpisheh et al., 2022). Short-chain fatty acids inhibit stem cell proliferation by inhibiting FoxP3-dependent histone deacetylase (Kaiko et al., 2016). Of course, colon crypts protect stem cells from metabolites derived from the microflora (Kaiko et al., 2016). Fecal short-chain fatty acids levels decline with age, which explains the increase in crypt stem cells seen with age (Salazar et al., 2019). Aging mice have been found to have decreased mucus barrier thickness, impaired M-cell antigen processing, and decreased number of specialized plasma cells (Sovran et al., 2019). Thus, as a result of age-related changes and stroke-induced dysbiosis, the microflora can penetrate the epithelium and initiate inflammation (Honarpisheh et al., 2022).

Aging is associated with remodeling of the immune system and low-grade chronic inflammation (inflammaging) resulting in uncontrolled immunosenescence and the development of chronic and degenerative diseases (Bleve et al., 2022). Immunosenescence is characterized by decreased immune efficacy against infection, impaired effector response to new antigens, increased autoimmunity and poor wound healing (Bleve et al., 2022). Inflammaging is often viewed as a consequence of inflammation often associated with lifestyle and unhealthy diet during the living period (Popa-Wagner et al., 2020), characterized by elevated levels of pro-inflammatory cytokines in the serum such as IL-1β, IL-6, IL-8, and TNF-α, and decreased levels of anti-inflammatory cytokines such as IL-10 (Conway et al., 2022).

Consistent with documented increased proliferation and tissue-redistribution of myeloid cells with aging, it was shown that peripherally sourced myeloid antigen-presenting cells significantly increase in the brain with advancing age (Honarpisheh et al., 2020a). The accumulation of myelocytes in the brain positively correlated with reduced *Akkermansia muciniphila*, age-related changes in the intestinal microflora and behavioral deficits (Honarpisheh et al., 2020a). Although the proportion of neutrophils in the peripheral circulation does not change, their functions such as phagocytosis, chemotaxis, and the production of reactive oxygen species are disturbed with age (Roy-O’Reilly et al., 2020; Conway et al., 2022). Aging exacerbates neutrophil pathogenicity in ischemic stroke (Roy-O’Reilly et al., 2020). Microbiota enhances neutrophil aging, and depletion of microflora reduced circulating older neutrophils and improved survival in animal model of endotoxin-induced septic shock (Zhang et al., 2015).

Age-related changes in the T cell population are characterized by a decreased number of naive T and Treg cells and increased CD8^+^ memory cells (McKenna et al., 2001). Age-related changes in B cells have been noted to be associated with a decrease in the precursors of the B cell lineage (McKenna et al., 2001). With age, peripheral B cell phenotypes shift from naive B cells toward B cells experiencing an antigen of limited clonal diversity with impaired ability to generate new high affinity immunoglobulins (Frasca and Blomberg, 2011). B lymphocytes accumulate with age in the blood and meninges and undergo an excessive proliferative response upon stimulation with toll-like receptor 9 and toll-like receptor 7 ligands (Rubtsov et al., 2011; Brioschi et al., 2021). Studies have shown that the marked shift in lymphocyte proliferation toward marrow cell proliferation with aging in pathogen-free mice is abolished (Krambs et al., 2021). There is an expansion of B cells in germ free mice, suggesting that microbiota signals suppress B lymphopoiesis with aging in mice (Krambs et al., 2021). Also, *Akkermansia muciniphila* supplementation in the accelerated aging model reduced the number of activated B lymphocytes in Peyer’s patches (van der Lugt et al., 2019). This suggests an involvement of the gut microbiota in the regulation of lymphocyte proliferation and suggests a possible therapy for lymphocyte dominant diseases in combination with approved B-cell depleting drugs (Honarpisheh et al., 2022).

The loss of microflora caused by antibiotics or in germ free models showed a harmful role of microflora in ischemic stroke (Pluta et al., 2021a, Honarpisheh et al., 2022). Lymphatic drainage of brain antigens decreases with age, suggesting that exposure of brain-derived antigens to border-bound macrophages and other antigen-presenting cells may be prolonged in old age compared to young stroke patients (Louveau et al., 2018; Rustenhoven et al., 2021; Honarpisheh et al., 2022). Metagenomic data indicate that increased abundance of *Akkermansia muciniphila* and butyrate production are responsible for the rejuvenation of the immune system seen in older mice (Shin et al., 2021). This suggests that the microflora can stimulate age-related inflammation of the central nervous system, regardless of the age of the host, and may contribute to the onset of neuroinflammation and reduce amyloid and tau protein pathology after ischemic stroke (Spychala et al., 2018; Kim et al., 2020; Pluta et al., 2021a). There is evidence that some Enterobacter species and fungi produce amyloid peptides or amyloid fibers associated with seed formation for amyloid aggregation in the brain (Lundmark et al., 2005; Zhou et al., 2012; Evans et al., 2015; Pluta et al., 2021a). Microbial amyloids induce nucleation of amyloid aggregates with high accumulation and an inflammatory response (Chen et al., 2016; Friedland and Chapman, 2017). In the absence of gut microflora, a reduction in amyloid accumulation was observed in the transgenic mice (Harach et al., 2017). It has also been noted that *in vitro* amyloid aggregation is inhibited by short chain fatty acids produced by the intestinal microflora (Ho et al., 2018). Additionally, bacterial endotoxin is responsible for inflammation of the central nervous system, causing the development of amyloid fibrils (Asti and Gioglio, 2014; Syed and Boles, 2014). Certain bacteria, such as *Escherichia coli*, produce amyloid, but the link between bacterial amyloid origin and post-ischemic neurodegeneration in the brain has not been definitively elucidated (Syed and Boles, 2014; Pluta et al., 2021a). Bacterial gram-negative lipopolysaccharide has been found to promote amyloid deposition in the brains of mice, adversely affecting cognition (Lee et al., 2008). It is not known how battery amyloids interact with other post-ischemic neuropathological elements such as post-translational changes in tau protein, production of β-amyloid peptides, neuroinflammation, and development of cerebral amyloid angiopathy (Kato et al., 1988; Pluta et al., 1994a, 2016a,b, 2018a,b, 2020a, 2021b, Pluta, 2000; Wen et al., 2007; Kocki et al., 2015; Ułamek-Kozioł et al., 2016, 2017, 2019; Khan et al., 2018; Hatsuta et al., 2019; Radenovic et al., 2020; Rost et al., 2022). As the permeability of the intestinal barrier increases with age, bacterial-derived amyloids enter the systemic circulation, aggravating inflammation in the brain and causing memory loss in mice (Jang et al., 2018; Pluta et al., 2020b). These data suggest that bacterial-derived amyloids play a significant role in enhancing the immune response and nucleation of amyloid aggregates in the brain following ischemia (Pluta et al., 2021a). Microbiome generated amyloid has a potential effect on amyloidogenesis in neurodegenerative disorders such as post-ischemic brain injury (Zhao and Lukiw, 2015; Pluta et al., 2021a). These suggestions have recently been strongly supported by *in vivo* and *in vitro* data (Lee et al., 2018; Zhao et al., 2018). By acting on brain gliosis, microbial amyloids are able to control brain neuroinflammation and levels of β-amyloid peptides (Zhao and Lukiw, 2015; Lee et al., 2018; Zhao et al., 2018). The altered bacterial flora influences the level of bacterial amyloid and metabolites in the blood and therefore may act as a trigger for the onset and exacerbation of neurodegeneration in the brain following ischemia (Pluta et al., 2020b, 2021a, Honarpisheh et al., 2022). Data from meta-analysis indicate that intestinal metabolites may increase inflammation, amyloid and tau protein aggregation in neurodegenerative diseases of the brain (Xu and Wang, 2016). The gut microbiota is involved in the secretion of over 100 metabolites, but their involvement in post-ischemic brain neuropathogenesis has not been definitively proven (Bostanciklioglu, 2019; Pluta et al., 2021a, Honarpisheh et al., 2022). It has been shown that valerian, isovaleric, isobutyric, butyric, propionic, acetic, and formic acids influence the development of neurodegenerative disorders by affecting the activity of astrocytes and microglia and reducing inflammation, amyloid and tau protein aggregation (Cummings et al., 1987; Macfarlane and Macfarlane, 2012). The host gut microflora has been found to influence microglial homeostasis in the brain, resulting in an immature microglial phenotype in germ-free animals, ultimately weakening the innate immune response (Erny et al., 2015). Attempts to re-colonize by the complex microflora partially restored the physiological features of the microglia (Erny et al., 2015). Taken together, these observations suggest that the gut microflora can control microglia maturation, function and activation, and therefore, in the case of impaired intestinal microflora, the maturation of the microglia and its phagocytic potential for amyloid and tau protein that accumulate after ischemia are limited (Kato et al., 1988; Pluta et al., 1994a, 2009, 2021a, Pluta, 2000; van Groen et al., 2005; Khan et al., 2018; Hatsuta et al., 2019; Honarpisheh et al., 2022). *In vitro* studies have shown that propionic, valeric and butyric acids inhibit the oligomerization of β-amyloid peptide 1-40 (Ho et al., 2018). After assessing the effect of intestinal microflora metabolites on β-amyloid peptide 1-42 aggregation, it was shown that only valeric acid inhibited the development of amyloid oligomers (Ho et al., 2018). Regarding the conversion of β-amyloid peptides into β-amyloid peptide fibrils, it was shown that both butyric and valeric acid inhibited the conversion of β-amyloid peptide 1-40 monomer to filamentous β-amyloid peptide (Ho et al., 2018). The dependence on the type of intestinal metabolite indicates that an increase in the amount of beneficial metabolites produced by the gut flora, especially anti-inflammatory bacteria, may aid in the removal of tau protein and amyloid from brain tissue following ischemia (Pluta et al., 2021a).

As much as 50% of the aging population suffer from chronic constipation and gastrointestinal problems (Honarpisheh et al., 2022). A 7-year clinical trial with over 3 million veterans found that a higher rate of constipation is independently associated with a higher risk of all-cause mortality, including ischemic stroke (Sumida et al., 2019). Investigations have shown that aging reduces the contractility of the intestinal smooth muscles (O’Mahony et al., 2002), their innervation (Phillips and Powley, 2007), and slows the intestinal transit time (Kim, 2017), suggesting age-related dysfunction of the autonomic nervous system that may be a consequence of changes in the microbiota and exacerbate complications after a stroke, such as a bowel obstruction.

Age-related changes in the functioning of the autonomic nervous system include diminished neurotransmitter activity, leading to impaired cerebral blood flow regulation and poorly controlled autonomic secretion, e.g., bladder function (Hotta and Uchida, 2010). Resting sympathetic tone increases with age, which is associated with increased blood levels of norepinephrine and decreased sensitivity of catecholamine and cholinergic receptors (Hotta and Uchida, 2010), indicating age-related dysfunction of the cholinergic anti-inflammatory pathway post-stroke (Honarpisheh et al., 2022). Fecal microflora transplantation from rats with spontaneous hypertension to rats with normal blood pressure increases sympathetic activity, blood pressure and inflammation of the central nervous system (Toral et al., 2019), suggesting that the microflora may mediate sympathetic pathophysiology observed with aging and post-ischemic stroke (Honarpisheh et al., 2022).

The neurons of the intestinal nervous system undergo age-related neurodegeneration (Saffrey, 2013). The loss of intestinal neurons with age reduces the density of the nerve fibers in the intestinal mucosa (Peck et al., 2009). Aging gut neurons show swollen and dystrophic features, contain excessive accumulation of lipofuscin, and have a higher generation of reactive oxygen species in the muscle plexus (Jurk et al., 2012). Age-related reductions in intestinal glial cells, proportional to neuronal loss, have also been demonstrated (Phillips et al., 2004). Although there are communication pathways between the microbiota and the intestinal nervous system, the direct involvement of age-related dysbiosis in the neurodegeneration of the intestinal nervous system has not been definitively elucidated (Honarpisheh et al., 2022).

In summary, all components of the microbiota-gut-brain axis undergo significant age-related changes, many of which may be directly influenced or even stimulated by microflora (Honarpisheh et al., 2022). Given the above data and the fact that ischemic stroke is a disease of the elderly, studies of the pathophysiology and sequence of stroke must take into account age-related changes in the host and its microflora, including dysbiosis, gastrointestinal dysfunction, immune remodeling, and other existing pathologies of central nervous system and systemic such as diabetes, atherosclerosis, and obesity (Popa-Wagner et al., 2020; Honarpisheh et al., 2022).

## Influence of the gut microbiome on brain homeostasis and aging

It has been shown that, in addition to healthy neuronal development, there is increasing evidence that the physiological gut microflora also strongly influences brain function and its architecture under homeostatic conditions and during normal aging (Honarpisheh et al., 2022). It has been shown that the integrity of the blood-brain barrier in the hippocampus, striatum and frontal cortex is influenced by the physiological gut microflora (Honarpisheh et al., 2022). It was noted that germ-free mice have an increased permeability of the blood-brain barrier, a phenomenon that is already visible during embryonic development due to the weakening of tight junctions connections due to reduced occludin and claudin 5 activity (Braniste et al., 2014). Re-colonization of germ-free mice with physiological gut microflora or the use of the short-chain fatty acid, butyrate, restored the integrity of the blood-brain barrier. Recently, the host microbiota has been shown to control microglia maturation and the brain’s innate immune function (Honarpisheh et al., 2022). Germ-free adult mice show inhibition of microglia growth under homeostatic conditions compared to microglia in pathogen-free mice (Erny et al., 2015). In addition, it is known that short-chain fatty acids derived from microbes can cross the blood-brain barrier (Huuskonen et al., 2004). The oral administration of a mixture of butyrate, acetate and propionate stimulated the maturation of the microglial cells, indicating that they can directly affect the microglia (Erny et al., 2015). In addition, physiological microbiota have been shown to influence neurogenesis in the hippocampus of adult mice, leading to more immature neuronal cells in germ-free mice, but not in conventionally colonized mice (Marín-Burgin and Schinder, 2012; Ogbonnaya et al., 2015). Normal aging has been found to be characterized by a progressive decline in cognitive function associated with changes in the gut microflora of these individuals (Partridge, 2001; Claesson et al., 2011). It is still not fully understood how aging and dysbiosis of the intestinal microflora, the characteristic elements of aging, change the plasticity and homeostasis of immune cells in the brain. The plasticity of brain immune cells influences the homeostasis of brain parenchyma as well as diseases and is strongly dependent on the microenvironment and systemic factors. Blood metabolites and cytokines have been shown to be altered by intestinal dysbiosis leading to dysregulation of the peripheral immune system (Arpaia et al., 2013; Bachem et al., 2019; Lehallier et al., 2019). Brain immunity and central nervous system inflammation are also indirectly altered by dysbiosis via signaling metabolites (neurotransmitters) produced by the intestinal microflora (Table 1; Erny et al., 2015; Dinan and Cryan, 2017; Ma et al., 2019). Some of the major microbial metabolites (neurotransmitters) and their role in brain health are presented in Table 1. There is evidence to suggest that the gut microflora plays an important role in gut-brain communication, ultimately shaping the aging of the central nervous system while helping to maintain nervous system function in old age, or promoting pathology by placing the gut microflora as a judge of age-related neurological deterioration (Madison and Kiecolt-Glaser, 2021). Dysbiosis of the gut microflora can induce abnormal immune responses, which in turn disrupt systemic and local homeostasis of the host, and existing evidence points to the importance of the gut microflora in age-related central nervous system disease such as ischemic stroke (Pluta et al., 2021a, Honarpisheh et al., 2022). Various research systems have used the manipulation of the senile microbiome through fecal microbiota transplantation, administration of prebiotics and probiotics, and dietary interventions to reduce age-related dysbiosis (Holmes et al., 2020). Cellular indexing of transcriptomes and epitopes by sequencing revealed transcriptional changes among inflammatory Ly6C^+^ monocytes and innate lymphoid cells associated with the central nervous system (Honarpisheh et al., 2022). Demonstrating the plasticity of immune cells during aging and intestinal dysbiosis can help learn about the critical components that determine the brain’s immunity during aging, and determine the onset of age-related neurodegenerative disorders (Golomb et al., 2020). Studies on the effect of aging intestinal microflora on cognitive decline by transplanting fecal microflora from older to juvenile rats have shown that transplanting fecal microflora impairs cognitive behavior in young recipient rats, leading to reduced regional homogeneity in the medial prefrontal cortex and the hippocampus, decreased neurotrophic expression factor of brain origin, N-methyl-D-aspartate receptor NR1 subunit and synaptophysin in synaptic structures (Li et al., 2020). In addition, young rats showed elevated levels of oxidative stress and pro-inflammatory cytokines following fecal microflora transplantation, indicating that oxidative stress and neuroinflammation may underlie gut-related cognitive decline during aging (Schächtle and Rosshart, 2021).

**TABLE 1:** Neurotransmitters (metabolites) made by the intestinal microbiota and their role in the functioning of the brain.

Gut microbiota	Neurotransmitters	Research on	Participation in brain function	References
*Coryneform*, *Bacteroides vulgatus*, *Campylobacter jejuni*, *Lactobacillus*	Glutamate	Human/animal	Regulates learning, memory, and synaptic plasticity	Chang et al., 2020
*Lactobacillus*, *Bacillus*	Acetylcholine	Animal	Regulates memory, emotional personality, self-care ability, cognition, and social life ability.	Chen et al., 2017; Baxter and Crimins, 2018
*Lactobacillus*, *Escherichia*, *Streptococcus*, *Lactococcus*, *Bacillus*	Dopamine	Human/animal	Protects neuron loss, improves motor deficits, cognition, reduced stress and anxiety.	Holzer and Farzi, 2014; Koutzoumis et al., 2020; Liu et al., 2020
*Lactobacillus plantarum*, *Bifidobacterium adolescentis*	Gamma-aminobutyric acid	Human/animal	Regulates depression, anxiety, behavioral and cognitive functions.	Duranti et al., 2020; Yunes et al., 2020
*Akkermansia muciniphila*, *Lactobacillus plantarum DR7*	Serotonin and 5-hydroxytryptamine	Animal	Regulates learning, mood, cognition, and memory.	Liu et al., 2020; Yaghoubfar et al., 2020; Zaydi et al., 2020
*Escherichia coli*, *Morganella morganii*, *Lactobacillus vaginalis*, *Enterobacter aerogenes*	Histamine	Human/animal	Regulates depression-like behaviors and impaired sleep-wake cycle.	Barcik et al., 2016; Blasco et al., 2020; Yamada et al., 2020

In summary, these studies indicate a significant role of the intestinal microflora in the maintenance of neuronal homeostasis as well as in normal and pathological aging. A well-balanced equilibrium between the gut microflora and the host seems to be essential for maintaining the health of neuronal cells, while meaningful disturbances in this relationship can lead to the development of various mental and neurological disease entities.

## Post-ischemic pathology of the brain

Post-ischemic brain neurodegeneration in rodents and humans is characterized by the accumulation and deposition of misfolded amyloid in the form of amyloid plaques and the aggregation of misfolded tau protein in the form of neurofibrillary tangles, neuroinflammation as well as brain atrophy, ultimately leading to cognitive decline and dementia (Kato et al., 1988; Pluta et al., 1994a, 2009, 2018b, 2021b; Jendroska et al., 1995, 1997; Pluta, 2000; van Groen et al., 2005; Qi et al., 2007; Wen et al., 2007; Gemmell et al., 2012, 2014; Sekeljic et al., 2012; Khan et al., 2018; Hatsuta et al., 2019; Radenovic et al., 2020; Rost et al., 2022). Amyloid monomers aggregate into soluble, low molecular weight oligomers that spread throughout the brain and then aggregate into insoluble amyloid fibrils, gradually forming amyloid plaques (van Groen et al., 2005). Aggregation of the tau protein into intracellular neurofibrillary tangles is supported by various post-translational modifications such as hyperphosphorylation (Kato et al., 1988; Wen et al., 2007; Khan et al., 2018; Pluta et al., 2018b, 2021b; Hatsuta et al., 2019). An imbalance in the production and removal of amyloid from the brain following ischemia indicates that the brain favors the pathological process of amyloid aggregation (Pluta et al., 1994a, 2009; Pluta, 2000). Since the interstitial fluid, cerebral spinal fluid contributes to amyloid removal, amyloid and tau protein can be observed in both cerebral spinal fluid and peripheral blood serum (Pluta et al., 2011; Rickenbach and Gericke, 2022). In the brain, amyloid is locally degraded by enzymes in cells residing in the brain, including microglia and astrocytes, which help to reduce the amyloid load in the brain (Rickenbach and Gericke, 2022). Since the amyloid protein precursor is also present in the cell membranes of other organs and tissues, peripherally produced amyloid contributes to the total amyloid pool (Pluta et al., 1996; Rickenbach and Gericke, 2022). At the periphery, amyloid is cleared by monocytes, macrophages, and neutrophils through phagocytosis and endocytosis (Rickenbach and Gericke, 2022). The brain amyloid load is also reduced by sequestration of soluble amyloid in the blood by anti-amyloid antibodies and low density lipoprotein receptor-related protein 1 through a peripheral “sink” effect (Rickenbach and Gericke, 2022).

Experimental data show the involvement of the adaptive immune system in the post-ischemic brain, which is associated with an increase in CD4 and CD8 of T cells in the brain (Sekeljic et al., 2012). To date, it is not fully understood whether the extravasation of T lymphocytes to the brain is the result of ischemic damage to the brain-blood barrier and microbleeds, or whether T lymphocytes are specially recruited to the brain by recognizing brain-derived antigens (Rost et al., 2022). Studies in murine models of β-amyloidosis showed that although microhemorrhages contributed to non-specific T cell extravasation, specific infiltration of T cells into the parenchyma of the brain was mainly associated with amyloidosis and tau protein pathology (Rickenbach and Gericke, 2022). Alternatively, new T-cell antigens, so-called neo-epitopes, can be created by splicing mechanisms during processing in antigen presenting cells (Rickenbach and Gericke, 2022).

The immune receptor TREM2 is required for microglia responses, including microglia accumulation in proximity to amyloid deposition and amyloid plaque phagocytosis. Treatment with an anti-TREM2 agonist antibody has been shown to promote microglia activation and induce phagocytosis of amyloid in murine models of β-amyloidosis, reducing the amount of amyloid plaques and neuronal damage (Wang et al., 2020). Anti-amyloid and tau protein autoantibodies are naturally present in the serum and cerebrospinal fluid of Alzheimer’s disease patients (Rickenbach and Gericke, 2022) and should be expected to after cerebral ischemia. A potential neuroprotective role and a relationship between autoantibody levels and the progression of brain neurodegeneration following ischemia are to be expected.

## Ischemic stroke and the intestinal microflora

Currently, ischemic stroke is the leading cause of death and long-term disability worldwide (Benjamin et al., 2018). A stroke is caused by a blood clot or embolism in the arteries that supply the brain with blood (Pluta et al., 1994b, Benjamin et al., 2018). Ischemic stroke survival has dramatically improved with advances in emergency treatment with intravenous thrombolysis or intravascular thrombectomy (Powers et al., 2019). Due to the exclusion criteria for post-stroke time, risk of hemorrhage, and the need for specialized resources, less than 10% of stroke patients receive this treatment (Rinaldo et al., 2019). Poor long-term functional outcomes in stroke survivors are a major factor in lowering quality of life and increasing the socioeconomic burden post-stroke (Gbd 2013 DALYs and Hale Collaborators et al., 2015).

The post-stroke infection rate is around 30%, and the development of post-stroke infection is associated with poor recovery outcomes (Popović et al., 2013). About 50% of post-stroke patients also experience gastrointestinal complications, including gastrointestinal bleeding, dysbiosis and loss of peristalsis resulting in longer hospital stays, higher mortality, and poorer neurological outcomes (Camara-Lemarroy et al., 2014). Signaling phenomena caused by stroke do not end with disturbing gastrointestinal homeostasis and microflora (Honarpisheh et al., 2022). The signaling wave from the gastrointestinal tract spreads toward the brain, causing the subacute and chronic phase of the response to the stroke (Honarpisheh et al., 2022). Above, it was shown how dysbiosis and aging affect microbiota-gut-brain axis signaling, playing a detrimental but potentially reversible role in stroke neurological outcomes. Although stroke is a disease of aging, but the majority of preclinical research has been conducted in young animals.

Numerous studies have shown that infarct volume is smaller after reversible middle cerebral artery occlusion in older animals compared to matched young, even with the same duration of ischemia and post-ischemic survival (Shapira et al., 2002; Liu et al., 2009; Liu and McCullough, 2011; Sieber et al., 2011). Despite having a smaller volume of infarction, older rodents have poorer neurological outcomes and higher mortality compared to younger rodents (Shapira et al., 2002; Liu et al., 2009; Liu and McCullough, 2011; Sieber et al., 2011). On the other hand, the few studies using photochemically induced local cerebral ischemia have shown larger infarcts in older animals (Kharlamov et al., 2000; Wang et al., 2003). In addition to age, the infarct volume after an experimental stroke is influenced by many factors, including the species studied, reperfusion after ischemia vs. permanent ischemia, and existing differences in collateral circulation. Thus, in most studies that used the reversible middle cerebral artery occlusion model, older rodents had less severe brain edema and smaller infarcts, but showed higher mortality and poorer neurological outcomes post-stroke compared to young controls (Shapira et al., 2002; Liu et al., 2009; Liu and McCullough, 2011; Sieber et al., 2011).

Stroke risk factors include both intra- and inter-individual changes in the intestinal microflora (Fei et al., 2019). It has been shown that a metabolic shift of tryptophan metabolism from a microbiota-regulated indole pathway toward a host-regulated kynurenine pathway may have a detrimental effect on the development of cerebral atherosclerosis, a major risk factor for stroke (Wikoff et al., 2009; Cason et al., 2018).

Risk factors for ischemic stroke such as obesity and diabetes (Popa-Wagner et al., 2020) are transmitted through fecal microflora transplantation (Honarpisheh et al., 2022). Targeting the microflora to reduce metabolic disturbances can be used for the clinical control of metabolic risk factors for ischemic stroke. Dysfunction of the microbiota-gut-brain axis contributes to arterial hypertension through metabolites, bacteria in the circulatory system and autonomic system dysregulation (Richards et al., 2022). Evidence suggests that the gut microflora may independently contribute to the development of significant risk factors for ischemic stroke which are modulated or transmitted by the gut microflora (Honarpisheh et al., 2022).

A significant proportion of older post-stroke patients are weak, linked to cerebrovascular disease, and weakness predicts shorter post-stroke survival (Palmer et al., 2019). General weakness is characterized by decreased grip strength, reduced energy, slowness of movement, decreased physical activity, and unintentional weight loss (Fried et al., 2001). A specific feature of the weakness is the loss of microflora diversity and taxonomic changes, including an increase in the number of *Eubacterium dolichum* and *Eggerthella lenta*, a decrease in the number of *Faecalibacterium prausnitzii*, a loss of butyrate producers, a decrease in the metabolism of tryptophan and an increased production of peptidoglycans and lipopolysaccharides (Jackson et al., 2016). There are strong positive associations between microflora diversity and less fragility, which improves immune function and increased cognitive performance (Claesson et al., 2012). Oral administration of *Akkermansia muciniphila* changed the aging phenotype in the intestines of older mice and prolonged health, as evidenced by the recovery of atrophied muscles (Shin et al., 2021). These observations suggest that identifying microbial changes during aging could be the basis for developing bacteriotherapy for healthy aging and recovery from stroke. A placebo-controlled clinical trial in 55 patients with an overgrowth of bacteria in the small intestine showed that a 4-week treatment with fecal transplant capsules prepared from healthy donors resulted in a significant increase in bacterial diversity and a reduction in gastrointestinal symptoms (Xu et al., 2021a).

An ischemic stroke is a type of focal cerebral ischemia with an ischemic core where neurons die quickly and there is a penumbra on the periphery of that core that remains at risk but can be saved. A damaged core initiates inflammation and apoptosis through glutamate excitotoxicity, changes in calcium levels and oxidative stress (Spychala et al., 2017). Disruption of cerebral blood flow activates endothelial cells and initiates the coagulation cascade and vessel spasm (Pluta et al., 1994b, Wisniewski et al., 1995). Once the blood-brain barrier is opened after stroke, blood-borne immune cells are transmigrated within minutes into ischemic focus (Yilmaz and Granger, 2010; Iadecola et al., 2020).

Age influences the processes involved in the early response to stroke, including the age-related increase in the level of coagulation factors in the blood which leads to procoagulation in the elderly and increases the risk of post-stroke complications (Tschan and Bolliger, 2021). Hemorrhagic transformation of stroke is more common in older mice than in young mice compared to the size of the infarction, which is related to the earlier influx of neutrophils into the aging brain that produce metalloproteinases and this leads to damage of the blood-brain barrier (Ritzel et al., 2018). Subsequently, the recruitment of phagocytes and granulocytes initiates the removal of damaged neuroglia after ischemia (Schilling et al., 2003). Aging alters the immune response to ischemic stroke, so manipulating peripheral immunity by acting on the bone marrow or the microbiota can reverse the age-related response to stroke (Ritzel et al., 2018; Iadecola et al., 2020).

Complete removal of microglial cells worsens post-stroke outcomes, suggesting that microglia plays a beneficial role after stroke, possibly by suppressing neuroinflammation caused by neuroglial damage (Jin et al., 2017). Aging has a significant influence on the microglia response after a stroke (Ritzel et al., 2018; Honarpisheh et al., 2020b, Conway et al., 2022). Aging microglial cells produce less TNF-α but more ROS than young microglia, and old mice with a young bone marrow transplant showed a reduced microglia response after stroke (Ritzel et al., 2018). Also, transplantation of the post-stroke fecal flora of young healthy mice to older mice reduced microglia activation (Lee et al., 2020). These observations indicate that the age-dependent role of the microglia after stroke may be influenced by the manipulation of the intestinal microflora.

## Congenital immune response following a stroke of old age

Increased neutrophil infiltration into the brain following stroke was noted in older animals (Ritzel et al., 2018). Aging neutrophils have decreased phagocytosis but produce more reactive oxygen species and metaloproteinaze-9 following stroke (Ritzel et al., 2018; Roy-O’Reilly et al., 2020). The response of macrophages related to the brain environment, blood-derived monocytes and dendritic cells is among the early responses to ischemic stroke. Recruitment of monocyte-derived macrophages via the C-C chemokine receptor 2 is protective in the early stages after stroke, contributing to phagocytic activity and debris removal, however, their continued presence contributes to chronic neuroinflammation (Sekeljic et al., 2012; Radenovic et al., 2020; Honarpisheh et al., 2022). The bone marrow and spleen are the main sources of monocytes and have been shown to have a significant influence on the development of systemic inflammation, including enteritis (Chauhan et al., 2018). But splenectomy protects older mice from ischemic stroke injuries (Chauhan et al., 2018). The observed elevated expression of toll-like receptor 4 on peripheral blood monocytes of stroke patients aggravated stroke and inflammatory lesions, and the same monocytes were also involved in systemic inflammation following stroke (Yang et al., 2008). It is not fully understood whether age-related changes in the microbiota affect neutrophils and monocytes of peripheral origin following stroke. Dendritic cells have been shown to arrive early in the focus of stroke and remain there for several days (Felger et al., 2010). Studies have shown that, under the control of the gut microflora, dendritic cells are involved in the development of inflammation by secreting IL-23, inducing IL-17 expression by γδ T cells, and promoting neutrophil infiltration (Benakis et al., 2016). The data show that the number of antigen presenting cells in the brain before stroke is significantly higher in older mice and is correlated with specific age-related changes in microflora (Honarpisheh et al., 2020b). Studies have shown that more neutrophils are moving into the older brain during 3 days after the stroke (Ritzel et al., 2018). The data indicate that the composition of the myeloid populations in the bone marrow of the skull bones changes with age with a significant increase in the number of neutrophils in the aging skull, which may explain the increased influx of neutrophils into the stroke area in older mice compared to young mice (Cugurra et al., 2021).

## Adaptive immune response following a stroke of old age

As a result of an episode of reversible cerebral ischemia with inflammation, the blood-brain barrier, the lymphatic system of the meninges and the blood-cerebrospinal fluid barrier are damaged, which allows the passage of antigens and cytokines of brain origin into the systemic circulation (Bernardo-Castro et al., 2020; Iadecola et al., 2020). In addition, an increased number of circulating lymphocytes has been associated with a higher risk of recurrent stroke and its mortality (Nadareishvili et al., 2004). Increased numbers of T helper 17 cells in the blood are associated with increased cognitive deficits after stroke, suggesting an involvement of antigenic lymphocyte activation in the chronic sequelae of stroke, such as cognitive decline and autoimmunity (Yin and Li, 2011; Swardfager et al., 2014). Indeed, autoreactive B and T lymphocytes are present in the stroke focus as early as 4 days after stroke, while circulating lymphocytes from patients collected 2 weeks after stroke show stronger immunoreactivity to brain antigens than those collected from the control group (Youngchaiyud et al., 1974).

More research is needed to determine whether this age-dependent heterogeneity of myeloid cells and lymphocytes differently contributes to the post-stroke response (Singh et al., 2018). Their proximity, temporal involvement, and specific cellular regulation by the gut microbiota following acute cerebral ischemia, combined with the slower lymph clearance seen with aging, may result in prolonged exposure to new brain antigens, which may contribute to an increased incidence of autoimmunity and long-term, irreversible cognitive decline observed in patients after ischemic stroke (Doyle et al., 2015; Ma et al., 2017; Javidi and Magnus, 2019).

## Age worsens post-stroke dysbiosis

The systemic phenomena are involved in post-stroke injury: systemic immune answer, e.g., migration of monocytes and neutrophils in reply to brain signals, activation of the hypothalamic-pituitary-adrenal axis affecting the intestinal barrier function, and activation of the sympathetic system and loss of parasympathetic drive, disrupting intestinal motility by intestinal nervous system and gastrointestinal secretions by enteroendocrine and Paneth cells (Honarpisheh et al., 2020a,b). These phenomena trigger after stroke dysbiosis, often superimposed on age-related dysbiosis and immunosenescence (Benakis et al., 2016; Xia et al., 2019; Lee et al., 2020). Dysbiosis following stroke exacerbates systemic inflammation, by creating a vicious cycle of damage between the brain and gut.

## Experimental research on the role of aging gut microflora in stroke

In preclinical investigations, the complete absence of microflora in germ-free and antibiotic-treated mice leads to poorer post-stroke outcomes (Winek et al., 2016). Moreover, pre-stroke bacterial colonization from non-stroke-specific pathogen-free mice into germ-free mice increases microglia/macrophage counts and brain cytokines (IL-1β and TNF-α) and reduces infarct volume in germ-free mice, indicating that pre-stroke microbiota from young healthy donors may play a neuroprotective role post-stroke (Singh et al., 2016, 2018). On the other hand, transplanting pre-stroke fecal microflora from specific pathogen-free mice with stroke increased infarct volume and worsened post-stroke neurological deficits in germ-free mice (Singh et al., 2016, 2018). Consequently, transplantation of pre-stroke fecal microflora from stroke patients to antibiotic-treated mice increased infarct volume and neurological deficits in recipient mice (Xia et al., 2019). These results suggest that pre-stroke transplantation with microflora from healthy donors has a neuroprotective effect, but transplantation with age-related dysbiotic microbiota and from patients with prior stroke has a detrimental effect on the outcome of stroke (Honarpisheh et al., 2022). The effect of a prior stroke on the immune response to a next/new stroke is clinically important and requires further research. Importantly, older mice not only have an underlying dysbiotic state, but are also more prone to stroke-induced dysbiosis, as manifested by increased intestinal permeability and subsequent septicemia due to bacterial translocation (Table 2; Crapser et al., 2016). Aging mice receiving fecal transplant prior to stroke from young donors have better survival rates, decreased circulating cytokines, and better behavioral outcomes even though infarct size has not changed after stroke (Table 2; Spychala et al., 2018). This suggests that rejuvenating the microflora in elderly patients at risk of stroke may be a promising preventive therapy. It should be emphasized that, with the exception of a few studies, most of the preclinical studies were carried out in young mice, and microflora transplants were performed before stroke, which significantly reduces the translational value of the results (Lee et al., 2020).

**TABLE 2:** Experimental studies on the role of aging gut microflora in stroke.

Age of animals (months)	Animals	Type of ischemia	Key observations	References
18–20	Mouse	MCAO	*Bifidobacterium longum*, *Clostridium symbiosum*, *Faecalibacterium prausnitzii* and *Lactobacillus fermentum* producers of short-chain fatty acids used for transplantation alleviated post-stroke neurological deficits and inflammation and increased concentrations of short-chain fatty acids in the intestines, brain, and blood in old mice after stroke.	Lee et al., 2020
18–20	Mouse	MCAO	Studies have shown that both directions of the gut-brain axis are functional in the context of ischemic stroke in old mice. Stroke has been shown to cause additional dysbiosis in old mice. In addition, the age of the gut microflora influenced behavioral outcomes and recovery post-stroke. After stroke, older mice go into a vicious circle that slows down the mechanisms involved in recovery and strengthens those mechanisms that worsen outcomes, including severity of systemic inflammation.	Spychala et al., 2018
18–20	Mouse	MCAO	In the old mice, the stroke induced intestinal permeability and translocation of bacteria, and these mice were unable to recover from the infection. As a consequence, these mice developed a post-stroke septic response characterized by persistent and severe hypothermia, immune system dysfunction and weight loss.	Crapser et al., 2016

MCAO, middle cerebral artery occlusion.

## Clinical studies on the role of aging gut microflora in stroke

Clinical trials have shown a reduction in the diversity of bacteria in samples taken from stroke patients compared with healthy patients (Yin et al., 2015; Haak et al., 2021). In Japanese stroke patients, an increase in *Lactobacillus ruminis* and a decrease in acetate and valerate were found to positively correlate with markers of systemic inflammation after stroke (Yamashiro et al., 2017). Other studies found that *Oscillospira*, *Enterobacteriaceae*, and *Parabacteroides* were enriched in stroke patients, while *Faecalibacteriu*m, *Roseburia*, and *Prevotella* were reduced (Xia et al., 2019). Moreover, it was shown that higher *Enterobacteriaceae* numbers were correlated with poor neurological outcomes (Xu et al., 2021b).

## Conclusion

The effect of the aging gut microflora on ischemic stroke is unique as it affects both the risk and outcome of a stroke (Panther et al., 2022). Clinical studies have shown that patients with the most known risk factors (e.g., unhealthy eating habits, diabetes, obesity, and stimulants) (Popa-Wagner et al., 2020) of ischemic stroke have a significantly altered composition of the intestinal microflora (Zeng et al., 2019). In addition, it has been proven that high-risk patients have a reduced number of butyrate-producing bacteria and a lower concentration of butyrate in the stool (Honarpisheh et al., 2022). Also clinical investigations performed following ischemic stroke have shown reduced levels of short-chain fatty acids in stroke patients compared to healthy persons (Tan et al., 2021). Fecal short-chain fatty acids levels are inversely related to functional outcomes ninety days after stroke (Tan et al., 2021; Honarpisheh et al., 2022). However, it should be remembered that these changes may rather be a secondary phenomenon co-occurring with the basic phenomenon (stroke), but not significantly influencing its course (Honarpisheh et al., 2022). An inadequate diet and limited mobility after an ischemic stroke can lead to a poor microbiome. Nevertheless, these evidences suggest that not only the gut-brain axis is an important risk and outcome factor for stroke, but that metabolites such as short-chain fatty acids, glutamate, acetylcholine, dopamine, GABA, serotonin, and histamine may be important functional components in this relationship (Table 1). The main difference between the microbiota of young and old mice is the reduction of bacteria that produce short-chain fatty acids as neurotransmitters (Coman and Vodnar, 2020; Honarpisheh et al., 2022). Old mice that received a fecal transplant from young mice after occlusion of the middle cerebral artery showed better functional regeneration than those that received a fecal transplant from older mice (Lee et al., 2020). Changes in specific types of bacteria are also consistent with the observation that different experimental antibiotic treatment regimens have different effects on stroke outcome. Treatment of mice with clavulanic acid and amoxicillin reduces the infarct volume post-stroke, while a broad-spectrum cocktail of ciprofloxacin, ampicillin, metronidazole, imipenem and vancomycin reduces survival (Benakis et al., 2016; Winek et al., 2016).

Also these observations indicate that aging alters the innate and adaptive immune response following stroke with increased neutrophil infiltration, prior involvement of peripherally derived antigen presenting cells, and a potentially different proportion of cranial bone marrow-derived immune cells compared to peripheral lymphoid immune cells. It can be concluded that the age-related decrease in the number of protective bacteria, e.g., *Akkermansia muciniphila*, may lead to disturbances in the intestinal barrier function. Thus, γδ T cells move into the aging brain after stroke due to increased blood-brain barrier permeability and the damaged intestinal barrier as a result of stimulation by activated dendritic cells, increasing the production of IL-17, ultimately leading to additional recruitment of neutrophils from the plasma or cranial bone marrow. Research indicates that it is not only the presence, absence, or complete microbial load that governs the gut-brain axis, but rather the abundance and interactions between bacterial groups are important. Future research is needed to elucidate the role of age-related changes in the gut microbiota in cell-specific immune responses following stroke and in the long-term effects of stroke such as cognitive decline with development dementia.

## Author contributions

RP and MJ: idea of the manuscript preparation and editing. SJ: searching for literature and preparation of the tables. SC: preparation and editing of the manuscript. All authors contributed to the article and approved the submitted version.
